# Supplementary material for: RNA Interference by Ingested Dsrna-Expressing Bacteria to Study Porphyrin Pigmentation in Crassostrea gigas
Source: Int J Mol Sci. 2021 Jun 6;22(11):6120. doi: 10.3390/ijms22116120 (PMC8201132; doi:10.3390/ijms22116120)
Supplement: Supplementary file 1 [file ijms-22-06120-s001.zip › ijms-1150424-supplementary.pdf]

Supplementary Figures

Figure S1. qPCR assay for gene expression levels of *CgALAS, CgALAD, CgPBGD, CgUROS, CgUROD* transcript in individual oyster. Expression levels were normalized to *EF1* and gene expression levels are given as relative levels of RNAi-treated oysters against control oysters. Asterisks (*) indicate oysters showing the newly deposit shell.

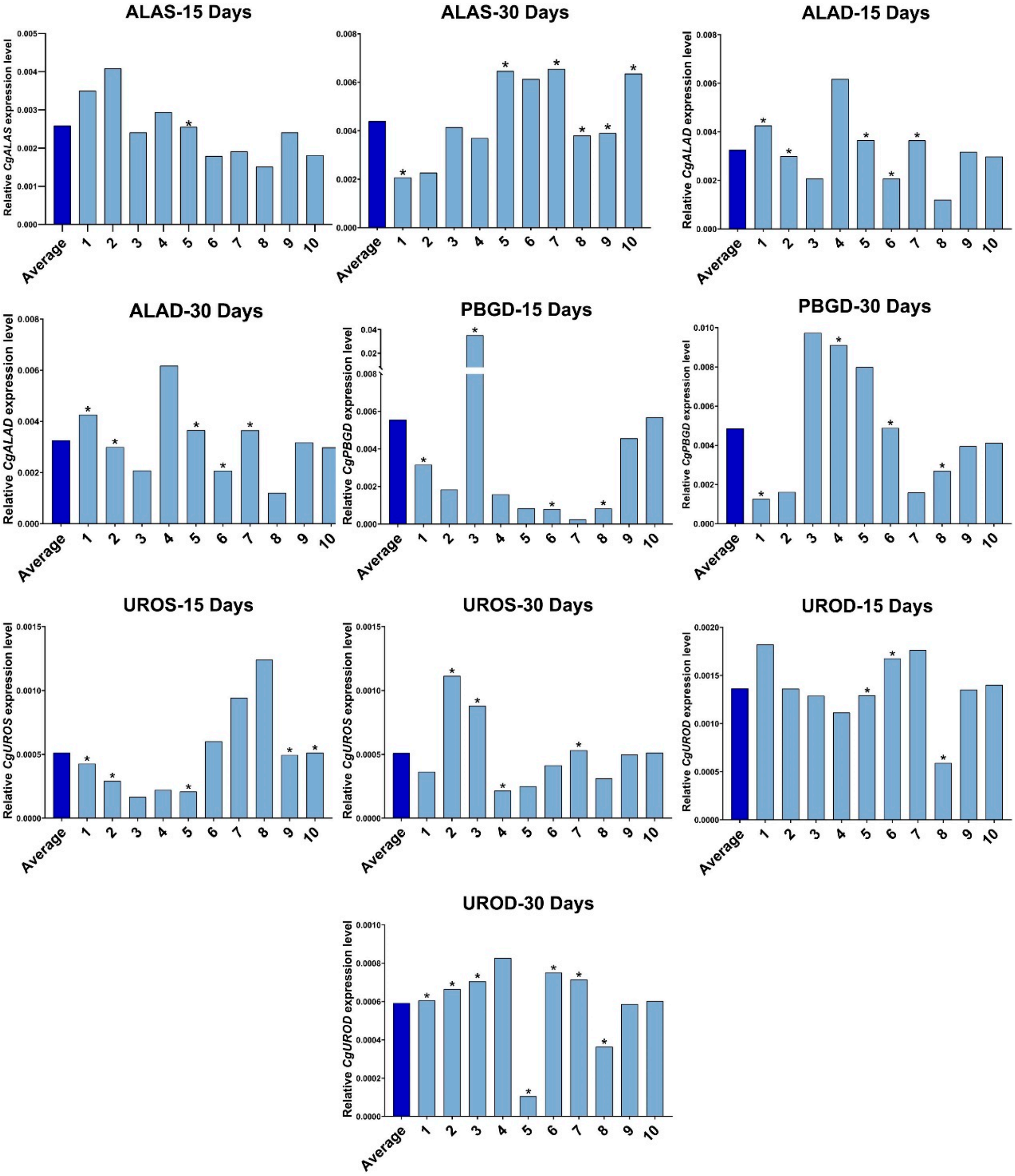

Supplementary Tables

Table S1. Probabilities for two-tailed, paired t tests for differences in gene expression levels among control and experiment groups of *C.gigas*. The *P* values from a t-test demonstrated **P* < 0.05, ** *P* < 0.01 or *** *P* < 0.0005.

***P* value**	**15 Days**	**30 Days**
ALAS versus EGFP	0.0476*	0.2096
ALAD versus EGFP	0.5633	0.9514
PBGD versus EGFP	<0.0001****	0.2155
UROS versus EGFP	0.1130	0.3917
UROD versus EGFP	<0.0001****	0.0008***

Table S2. Probabilities for two-tailed, paired t tests for differences in *L**a*b** value among control and experimental groups of *C.gigas.* The *P* values from a t-test demonstrated **P* < 0.05, ** *P* < 0.01, *** *P* < 0.0005 or **** *P* < 0.0001.

***L**a*b** comparisons**					
*L** EGFP Versus	*L** ALAS	*L** ALAD	*L** PBGD	*L** UROS	*L** UROD
	0.0856	0.1688	0.0006***	0.2798	0.9344
*a** EGFP Versus	*a** ALAS	*a** ALAD	*a** PBGD	*a** UROS	*a** UROD
	<0.0001****	0.1158	<0.0001****	0.1125	0.3767
*b** EGFP Versus	*b** ALAS	*b** ALAD	*b** PBGD	*b** UROS	*b** UROD
	0.0082**	0.9159	<0.0001****	0.8065	0.2639
